# Nanofabrication for all-soft and high-density electronic devices based on liquid metal

**DOI:** 10.1038/s41467-020-14814-y

**Published:** 2020-02-21

**Authors:** Min-gu Kim, Devin K. Brown, Oliver Brand

**Affiliations:** 1 School of Electrical and Computer Engineering, Georgia Institute of Technology, Atlanta, GA 30332 USA; 2 Institute for Electronics and Nanotechnology, Georgia Institute of Technology, Atlanta, GA 30332 USA; 3 Present Address: Department of Chemical Engineering, Stanford University, Stanford, CA 94305 USA

**Keywords:** Electrical and electronic engineering, Electronic devices, Nanoscale devices

## Abstract

Innovations in soft material synthesis and fabrication technologies have led to the development of integrated soft electronic devices. Such soft devices offer opportunities to interact with biological cells, mimicking their soft environment. However, existing fabrication technologies cannot create the submicron-scale, soft transducers needed for healthcare and medical applications involving single cells. This work presents a nanofabrication strategy to create submicron-scale, all-soft electronic devices based on eutectic gallium-indium alloy (EGaIn) using a hybrid method utilizing electron-beam lithography and soft lithography. The hybrid lithography process is applied to a biphasic structure, comprising a metallic adhesion layer coated with EGaIn, to create soft nano/microstructures embedded in elastomeric materials. Submicron-scale EGaIn thin-film patterning with feature sizes as small as 180 nm and 1 μm line spacing was achieved, resulting in the highest resolution EGaIn patterning technique to date. The resulting soft and stretchable EGaIn patterns offer a currently unrivaled combination of resolution, electrical conductivity, and electronic/wiring density.

## Abstract (web summary)

Though all-soft electronic devices with liquid metals are attractive due to the material’s compatibility with patterning methods, obtaining sub-micron patterns remains a challenge. Here, the authors report a hybrid lithography process to realize all-soft submicron-scale devices based on EGaIn.

## Introduction

Biological materials with dimensions ranging from the nanoscale, e.g. deoxyribonucleic acid (DNA), to the macroscale, e.g. tissues and organs, are generally curved, soft, and elastic, with low Young’s moduli *E* ranging from 100 Pa for brain tissue to 10 kPa for skin^1,2^. The resulting mismatch in mechanical properties at the interface of biology and traditional electronics often causes non-conformal contact, resulting in discomfort and performance degradation^3,4^. Furthermore, the large mechanical discrepancy at the cell-electrode interface affects multiple aspects of the cell’s behavior, such as its growth and differentiation, resulting in significant challenges for a variety of healthcare and medical devices^5,6^. In this respect, innovations in soft functional material synthesis and fabrication technologies have led to the development of integrated soft electronic devices for applications in human organs-on-chips as well as skin- or body-integrated electronics with material interfaces that are better matched from a mechanical properties point of view^6–9^. Biomedical applications, including platelet contraction (<5%), as well as intracranial (<7 kPa), intraocular (<5 kPa), and blood (<20 kPa) pressure applications, require submicron- or micron-scale soft sensing components with high-resolution sensing capabilities in the low strain and pressure regime^6,10–12^.

To enable soft and stretchable properties in electronic devices, both design and material strategies have been investigated^13^. In design-focused approaches, compliant two-dimensional (2D) serpentine or three-dimensional (3D) helical patterns are formed from solid metal thin films on soft substrates to endure mechanical deformation^14–16^. These engineered 2D/3D network architectures can interface with biological materials over large areas. However, their limited resolution (10 μm line width for 2D serpentine patterns^14^ and 50 μm line width for 3D helical patterns^15^) is one of the drawbacks for the fabrication of submicron- or nanoscale devices. Advanced nanoprinting techniques, such as nanotransfer printing, can create complex patterns with tens of nanometer resolution using high-resolution stamps fabricated by electron-beam lithography (EBL) or laser interference lithography (LIL)^16–18^. However, these printed nano patterns are not compliant structures, and the nanotransfer process is also limited to print on rigid substrates only. Moreover, the rigid metal patterns ultimately limit the strain the pattern can endure and lower the density of electronic components because of the use of space-consuming serpentine or helical wiring interconnections. On the other hand, material-focused approaches utilize elastic conductors based on conductive nanomaterials that are either embedded into polymer matrices or dispensed directly onto a soft substrate^19–21^. These printing approaches enable inexpensive fabrication processes for conductive circuits without the need of serpentine geometries, but the relatively low resolution (50–150 μm for conventional printing methods^19–21^) and low conductivity (<1 × 10^4^ S m^−1^ for single-walled-carbon-nanotube-doped elastic conductors^20^) of these conductors still limit their usability for high-density electronics integration. Overall, the major limitations for both approaches are patterning resolution, scalability, and resulting electronic density. In particular, scaling patterns down to submicron or even nanoscale dimensions is technically difficult using transfer printing techniques for serpentine or helical metal patterns or direct printing techniques for conductive nanomaterial networks^13–21^.

As an alternative to these more conventional approaches, the use of intrinsically soft, low melting temperature metals, such as gallium-based liquid metal (eutectic gallium-indium alloy, EGaIn, 75% Ga and 25% In, by weight), is a promising approach for all-soft electronic devices^22–24^. EGaIn offers a number of advantages, including a low melting temperature (MP < 15 °C), favorable mechanical stretchability (being a liquid, the stretchability is typically limited by the mechanical properties of the encasing material), thermal conductivity (*k* = 26.6 W m^−1^ K^−1^), and electrical conductivity (*σ* = 3.4 × 10^6^ S m^−1^)^22,23^. Under atmospheric oxygen level, a thin oxide layer (*t* ≈ 1–3 nm) is formed on the EGaIn surface, which allows EGaIn to be molded to elastomeric substrates^25^. Being a liquid-phase conductor with a brittle oxide layer on the surface, the shape of EGaIn-filled microchannels can be easily changed in response to applied mechanical forces, with a new oxide layer being formed instantaneously on the EGaIn surface after deformation, thus making it shape reconfigurable^22^. The moldable characteristics of EGaIn have resulted in the development of a broad range of patterning methods based on lithography-enabled stamping and stencil printing^26–33^, microfluidic injection^34–36^, as well as additive^37–39^ and subtractive^40–45^ patterning processes. However, creating fine and uniform EGaIn thin-film patterns using current EGaIn patterning technologies remains a major technical challenge because of the high surface tension of EGaIn (*γ* = 624 mN m^−1^)^23^. Using soft lithography^28,29^ or a selective wetting process^31^, the smallest EGaIn features demonstrated so far have a resolution of 2 μm with a thickness of ≈ 2 μm. Creating smaller and, especially, sub-micrometer EGaIn thin-film patterns remains challenging^22,28,29,31,39,46,47^.

For interfacing with individual cells, the ability to pattern sub-micrometer metallic structures embedded in soft substrates is, however, of significant interest. Considering the size of a single biological cell, such as platelets with a diameter of 2–3 μm, mechanotransducers should be manufactured with submicron-scale features and soft, biomimetic properties^1,48,49^. Existing fabrication technologies, including the transfer printing of compliant solid metal patterns^13–15^, nanoprinting^17,18^, direct printing of nanomaterials^19–21^, and EGaIn patterning^26–45^, are currently not suitable to fabricate such soft and stretchable electronic devices with submicron-scale resolution.

Building on our initial work^50^, this paper describes a nanofabrication strategy to create submicron-scale, all-soft electronic devices based on EGaIn. In particular, a hybrid lithography process is introduced that combines electron-beam lithography (EBL) for nano/microstructure fabrication with soft lithography for EGaIn transfer. This hybrid lithography process is applied to a biphasic structure, consisting of a metallic adhesion layer coated with EGaIn. The proposed hybrid fabrication approach enables high-resolution and high-density all-soft electronic devices, including passive electronic components, resistive strain sensor arrays, and microelectrode arrays. In particular, EGaIn thin-film patterning with feature sizes as small as 180 nm and 1 μm line spacing is demonstrated. The intrinsically soft EGaIn structures, patterned by the developed hybrid lithography technique, offer a currently unrivaled combination of resolution, electrical conductivity, and electronic/wiring density. Thanks to the intrinsically soft EGaIn properties, the fabricated soft devices can endure mechanical deformation up to 30%, while maintaining electrical functionality.

## Results

### Nanofabrication based on hybrid lithography process

**Fig. 1:**
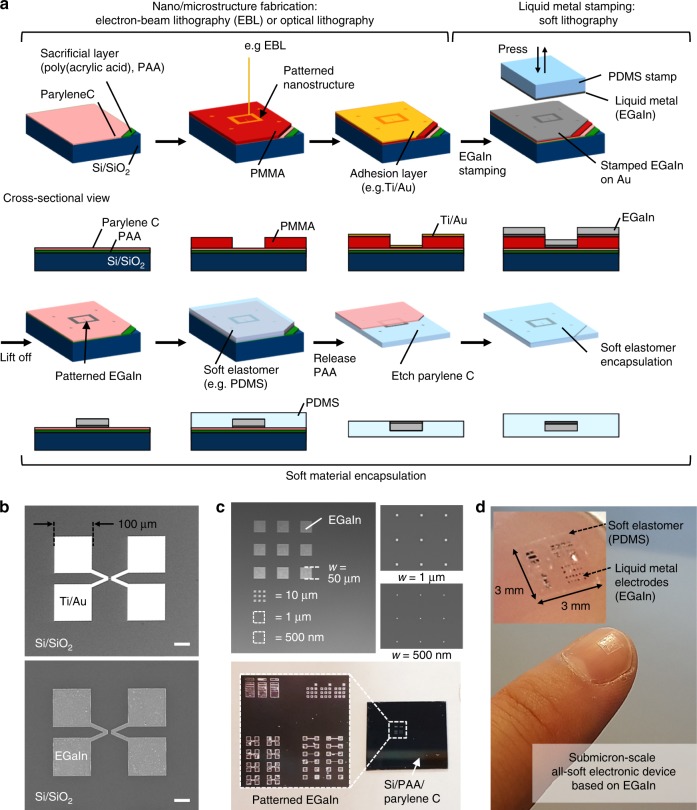
Nanofabrication process based on hybrid lithography for submicron-scale EGaIn patterning. **a** Hybrid lithography process combining electron-beam lithography and soft lithography for fabrication of submicron-scale, soft electronic devices based on EGaIn. **b** Patterned Au (top) and EGaIn on Au (bottom) structures. Scale bars indicate 40 μm. **c** Patterned EGaIn square-shaped dot arrays with various dot dimensions from 50 μm down to 500 nm, and **d** soft material encapsulation and release process of the EGaIn structures.

Figure 1a shows a schematic of the investigated nanofabrication process that combines EBL and soft lithography for submicron-scale EGaIn thin-film patterning. The fabrication process is comprised of three fundamental steps: nano/microstructure fabrication using EBL (or any other lithography technique able to pattern submicrometer features), EGaIn transfer using a stamping process, and soft material encapsulation and final release from the silicon (Si) carrier wafer. The process starts by spin-coating a water-soluble sacrificial material (poly(acrylic acid), PAA) on a silicon wafer at 2000 rpm for 30 s and baking the film at 100 °C for 60 s. On top of the PAA sacrificial layer, a 600-nm-thick parylene-C barrier film is deposited by chemical vapor deposition (CVD) in order to protect the underlying PAA during the subsequent EGaIn patterning as well as while releasing the fabricated soft electronic devices from the Si wafer after the soft material encapsulation. EBL is then used to pattern a spin-coated poly(methylmethacrylate) (PMMA) layer with a thickness between 300 nm and 1 µm. After exposure in the EBL tool (Elionix ELS G-100), the PMMA film is developed using a mixture of methyl isobutyl ketone (MIBK) and isopropanol with 1:1 ratio. Alternatively, other lithography processes with submicron resolution can be considered for this step. In the next step, a stamping process is used to transfer an EGaIn thin film onto the patterned PMMA structures. To improve the adhesion and uniformity of the stamped EGaIn on the parylene-C-coated substrate, a biphasic structure was adopted^51,52^. To this end, a thin metallic adhesion layer (such as Ti/Au, 5 nm/30 nm in thickness) is first deposited using electron-beam evaporation on the patterned PMMA nano/microstructures. The purpose of this metallic adhesion layer is to enhance the adhesion and wetting characteristics during the EGaIn stamping process while maintaining EGaIn’s electrical and mechanical properties. Then, a non-structured PDMS stamp is wet with EGaIn and gently pressed 2–3 times onto the Au-coated nano/microstructures, transferring a thin EGaIn film which forms an alloy with the underlying Au adhesion layer^51–53^. A PMMA lift-off process with acetone is then used to pattern the stamped EGaIn on Au. To highlight the impact of the Au adhesion layer on the EGaIn wettability, the EGaIn stamping process was carried out on patterned PMMA structures without and with the use of the Au adhesion layer (see Supplementary Fig. 1a–c). Without the adhesion layer (Supplementary Fig. 1a), the stamped EGaIn is not uniformly spread onto the patterned PMMA structure, resulting in non-uniform and rough EGaIn surfaces with EGaIn droplets as well as non-covered areas after PMMA lift-off. In contrast, by utilizing the Au adhesion layer during the EGaIn stamping process (Supplementary Fig. 1b, c), the stamped EGaIn uniformly spreads across the Au film and fills concave nano/micropatterns up to the designed PMMA thickness. Next, the remaining EGaIn structures are covered with a soft elastomer (e.g., poly(dimethylsiloxane), PDMS), and the fabricated devices are released from the Si carrier wafer by dissolving the sacrificial PAA layer in water for >6 h. Finally, the parylene-C barrier layer is etched using an oxygen plasma in a reactive-ion etching (RIE) system^27^, and the back side of the soft electronic device is sealed with a soft elastomer. It should be noted that optical lithography with a positive-tone photoresist can be utilized as well for the microstructure fabrication. Moreover, other lithography techniques able to pattern submicron-scale sacrificial structures, such as direct laser writing^54^ or 3D nanoprinting^55^, can be potentially utilized for cost-effective fabrication. The detailed fabrication process is described in the section Methods.

The EGaIn stamped on the Au adhesion layer demonstrated strong adhesion and uniform wetting and, therefore, could be successfully patterned using the PMMA lift-off process without any structural deformation. Figure 1b shows the patterned Au after lift-off without EGaIn stamping as well as the stamped and patterned EGaIn on Au. The images highlight the ability to pattern EGaIn using PMMA nano/microstructures written using EBL. Figure 1c, d depict the soft elastomer encapsulation and the release of the patterned EGaIn structures. In particular, Fig. 1c shows patterned square-shaped EGaIn dot arrays having dot dimensions from 50 μm down to 500 nm, while Supplementary Fig. 2 highlights 3D profile images of patterned EGaIn dot arrays of 1 µm and 500 nm width. EGaIn electrodes, including the square-shaped dot arrays, were patterned on the parylene-C barrier layer (Fig. 1c and Supplementary Fig. 3), encapsulated with PDMS and released from the Si wafer, as illustrated in Fig. 1a. Finally, Fig. 1d shows a fabricated all-soft electronic device based on EGaIn with PDMS encapsulation attached to the tip of a fingernail. The total thickness of the soft elastomer is controllable by selecting a proper target spin speed. The tested PDMS thickness was ≈ 50 μm, allowing for conformal wrapping on non-flat surfaces for skin- or body-integrated bioelectronic devices.

### Characterization of patterned liquid metal nano/microstructures

**Fig. 2:**
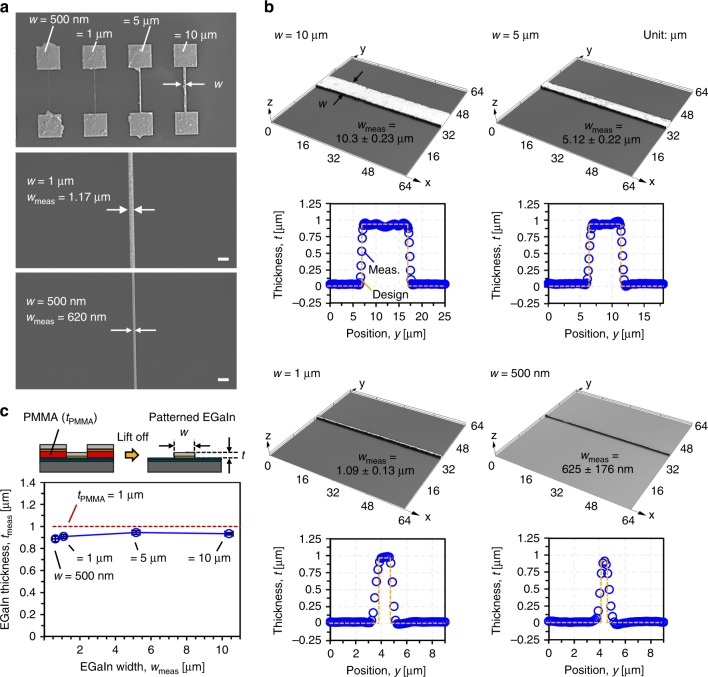
Patterned EGaIn nano/microstructures. **a** SEM images of EGaIn lines with widths from 10 µm to 500 nm and close-up of EGaIn lines with 1 µm and 500 nm width. Scale bars indicate 2 μm. **b** 3D images and cross-sections of EGaIn lines with width of 10 µm, 5 µm, 1 µm, and 500 nm. The blue symbols represent the measured profile, while the dashed yellow line indicates the designed cross-section. **c** Measured thickness of fabricated EGaIn lines as a function of line width. The x- and y-error bars represent the standard deviation. The thickness of the PMMA film (1 µm) is shown as dashed red line for reference.

To highlight the patterning capabilities of the hybrid lithography technique, EGaIn lines with different widths were fabricated using a 1-µm-thick PMMA layer. Figure 2a, b show SEM images and 3D profiles of the EGaIn lines with width ranging from 500 nm to 10 µm, respectively. As is highlighted in the 3D profiles in Fig. 2b, the patterned EGaIn lines exhibit sharp edges without EGaIn aggregation or loss during the lift-off process, which is attributed to the uniform and strong adhesion between Au and EGaIn^51,52^. During the PMMA lift-off process, all EGaIn on the top of the PMMA layer is washed away together with the PMMA. As a result, EGaIn only remains in areas where the underlying gold film is in direct contact with the Parylene-C layer after the lift-off process. The thickness of the resulting patterned EGaIn will be equal or less than the thickness of the PMMA layer. Figure 2c shows the measured EGaIn thickness after the lift-off process as a function of the patterned EGaIn line width and compares it to the designed PMMA thickness. The measured EGaIn thickness of ≈ 918 nm is slightly smaller than the PMMA thickness of 1 μm. Using a soft, deformable PDMS stamp for the EGaIn transfer, the stamped EGaIn uniformly spreads across the Au thin film while not covering all PMMA edges, which ultimately enables the EGaIn thin-film patterning using the PMMA lift-off process. The results of Fig. 2 indicate that the thickness of the resulting EGaIn film can be adjusted by controlling the thickness of the PMMA film. Also, the stamped EGaIn film uniformly fills the concave nano/microstructures.

**Fig. 3:**
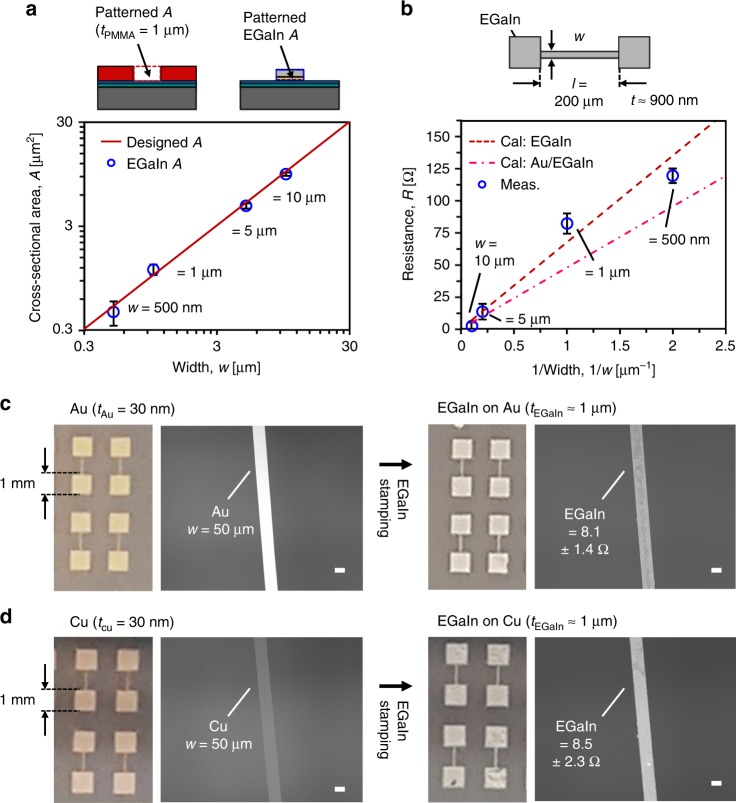
Characterization of patterned EGaIn nano/microstructures. **a** Measured cross-sectional area of EGaIn lines (symbols) with widths from 10 µm to 500 nm, compared to the designed cross-sectional area (solid line), as a function of the line width. The y-error bars represent the standard deviation. **b** Calculated (symbols) and measured resistance of 200-µm-long EGaIn lines as a function of line width. The y-error bars represent the standard deviation. The dashed line accounts for the resistance of the EGaIn film only while the dot-dashed line assumes a theoretical parallel circuit of the EGaIn film and the underlying Au adhesion layer. **c**, **d** EGaIn patterning characterization using different metallic adhesion layers, namely Au and Cu. **c** Patterned Au structures after lift-off process without EGaIn stamping and stamped and patterned EGaIn structures on Au. **d** Patterned Cu structures after lift-off process without EGaIn stamping and stamped and patterned EGaIn structures on Cu. Scale bars in **c** and **d** indicate 40 μm.

Figure 3a shows the measured cross-sectional area of fabricated EGaIn lines as a function of their measured line width and compare it with the designed cross-sectional area. The measured cross-sectional area of patterned EGaIn lines matches well with the designed value, which also confirms that EGaIn was uniformly patterned without EGaIn aggregation or loss during the lift-off process. Therefore, it is expected that the actual resistance of the patterned lines matches well with calculated values. To verify this, Fig. 3b depicts the measured resistance of fabricated EGaIn lines as a function of their line width for lines with 200 µm length, and compares these experimental values with values calculated based on the EGaIn structure dimensions alone as well as a theoretical parallel circuit formed by the combined Au and EGaIn structure. As expected, the measured resistance linearly scales with 1/width and the measured values closely match the calculated values based on the patterned EGaIn structure alone (assuming bulky resistivity of EGaIn), with <12% deviation. Thus, the effect of the thin Au adhesion layer used for the EGaIn transfer on the total resistivity of the combined structure appears to be minor. This indicates that the resistivity of the EGaIn dominates the resistance of the patterned biphasic EGaIn structures even in the presence of the Au adhesion layer^40^. To further demonstrate this, a different metallic adhesion layer, Cu, was adopted and the results compared to those obtained with the Au adhesion layer. Figure 3c shows patterned Au structures after the lift-off process without prior EGaIn stamping and the stamped and patterned EGaIn structures on the Au thin film, respectively. As expected, the resistance of EGaIn lines patterned on the Au adhesion layer agreed well with calculated values. Using the same resistor design and fabrication process, the EGaIn patterning process was performed using a thin Cu adhesion layer. Again, the stamped EGaIn uniformly spreads on the Cu film and no EGaIn aggregation or loss around the edges is observed during the lift-off process, as seen in Fig. 3d. The use of a thin Cu adhesion layer for the EGaIn patterning showed identical structures and electrical characteristics in comparison with the Au adhesion layer, which again confirms that the electrical performance of the biphasic EGaIn structures is mainly determined by the electrical properties of EGaIn as well as the geometry of the structures^40,52^.

**Fig. 4:**
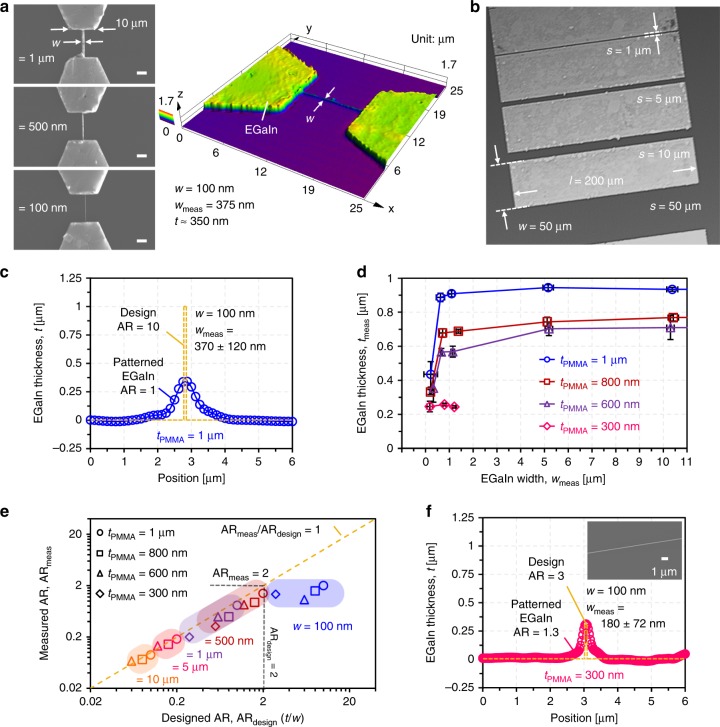
EGaIn patterning performance. **a** SEM images of EGaIn structures with designed line widths of 1 µm, 500 nm, and 100 nm on Si wafer as well as 3D profile of 100 nm EGaIn line fabricated using 1-µm PMMA thickness. Scale bars indicate 3 μm. **b** Patterned EGaIn test structures on Si wafer to evaluate achievable line spacing. **c** 3D cross-sectional profile of EGaIn line with designed 100-nm line width using 1 µm PMMA thickness. **d** Measured thickness of fabricated EGaIn lines as a function of designed EGaIn width for different PMMA thicknesses from 1 µm down to 300 nm. The x- and y-error bars represent the standard deviation. **e** Measured aspect ratio (AR) of EGaIn lines as a function of designed AR for PMMA thicknesses from 1 µm to 300 nm. **f** Measured 3D cross-section of EGaIn line with a designed width of 100 nm in the case of 300 nm PMMA thickness. The inset shows an SEM image of a fabricated 180-nm-wide EGaIn line.

The lateral resolution and line spacing achievable with the hybrid lithography technique has been investigated in more detail. Figure 4a, b show fabricated EGaIn structures used to evaluate the lateral resolution as well as the line spacing of the hybrid lithography technique, respectively. Using a PMMA film with 1 µm thickness, EGaIn lines with a designed width down to 500 nm at 1 µm spacing exhibit a constant ≈ 900 nm EGaIn thickness. On the other hand, EGaIn lines with a designed width of 100 nm collapsed downward after the lift-off process, yielding an effective line width of 370 ± 120 nm at a thickness of 350 nm, as shown in Fig. 4c. It is assumed that the high aspect ratio (AR) >10 of the initial design is responsible for this structural instability. At the same time, it is noted that the amount of EGaIn transferred is as designed, with the measured cross-sectional area of the patterned EGaIn line agreeing well with the designed value. Therefore, the aspect ratio of the PMMA structures is regarded as one of the most important design parameters when it comes to the structural stability of the fabricated EGaIn patterns. The smallest line spacing fabricated consistently between EGaIn features was 1 μm (Fig. 4b). To further investigate the impact of the AR on the achievable line width, PMMA film thicknesses from 1 μm down to 300 nm were used for EGaIn thin-film patterning. Figure 4d depicts the measured EGaIn thickness as a function of the designed EGaIn line width for different thicknesses of the PMMA resist. As expected, EGaIn lines with width >500 nm were patterned as designed without structural deformation because both designed and measured AR of the lines was <2 for the tested PMMA thicknesses ranging from 1 μm to 300 nm, as highlighted in Fig. 4e. In the case of lines with 100 nm width, patterned EGaIn structures for the PMMA thicknesses >600 nm collapsed downward because of the high designed AR > 6. However, this mechanical deformation can be minimized by adopting a 300-nm PMMA thickness. In this case, patterning of 180 ± 72 nm EGaIn thin-film lines with 250-nm line thickness can be achieved, as shown in Fig. 4f.

### High-resolution and high-density all-soft electronic devices

**Fig. 5:**
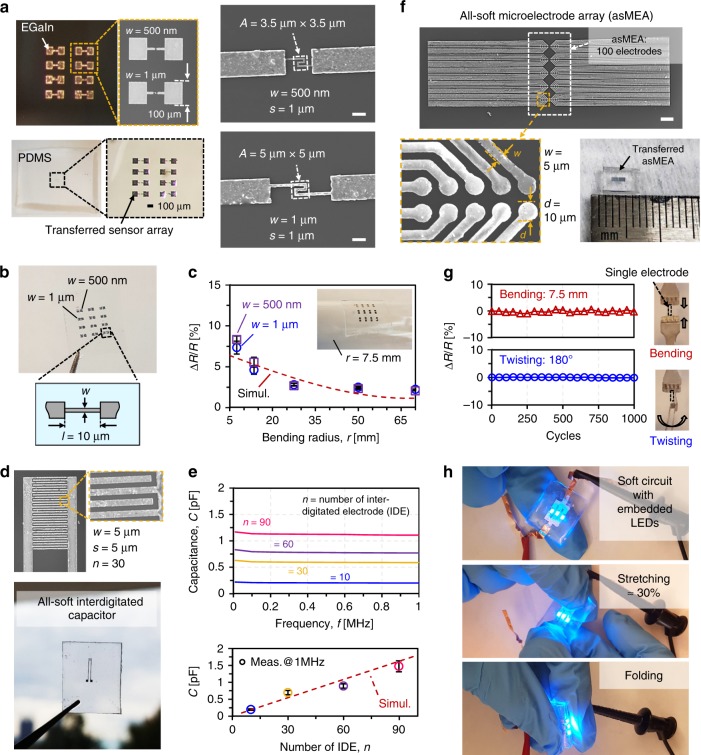
Fabricated all-soft electronic devices. **a** Resistive sensor arrays fabricated on a Si wafer with 500 nm line width and 1 µm spacing, and 1 µm line width and 1 µm spacing; Encapsulation with soft elastomeric material, such as PDMS, and release of soft resistive sensor array from the Si wafer. Scale bars indicate 5 μm. **b** Strain sensor array with straight-line resistors having 500 nm and 1 µm line widths and **c** measured and simulated relative resistance changes Δ*R*/*R* of the resistive strain sensors as a function of the bending radius. The y-error bars represent the standard deviation. **d** Interdigitated capacitors with 5 μm line width and spacing and **e** measured capacitance as a function of frequency (top) for different numbers of interdigitated electrodes (IDE) as well as measured and simulated capacitance as a function of the number of IDE (bottom). The y-error bars represent the standard deviation. **f** Microelectrode array with 100 electrodes (scale bar, 100 μm) and **g** measured relative resistance change as a function of the number of bending and twisting cycles (bending was performed around a cylinder with 7.5 mm radius, and twisting was performed to a 180˚ angle). **h** Fabricated soft circuit with three embedded LEDs under mechanical stretching and folding deformation.

To demonstrate the high-resolution patterning capability, all-soft passive electronic components, such as soft resistive sensor arrays and soft interdigitated capacitors, were investigated. Figure 5a shows a photograph of an array with eight EGaIn-based soft resistors, as well as an SEM image of two of the resistors with 500 nm and 1 μm line width, respectively. The patterned EGaIn resistor array was encapsulated with PDMS and subsequently released from the Si wafer, resulting in an all-soft, resistive sensing platform. To demonstrate its strain sensing capability, considering biomedical applications requiring high-resolution sensing devices in the low strain/pressure regime, the resistance of a soft resistive element was measured while bending the array around cylinders with known radii. Figure 5b shows the fabricated soft strain sensor array with 500 nm and 1 μm line widths, which have similar structures with Fig. 5a, but featuring straight-line resistors. In this particular experiment, a strain sensor array was characterized by attaching it to circular cylinders with bending radii ranging from 7.5 mm to 70 mm. Finite element simulations were performed in COMSOL Multiphysics (COMSOL Inc., Burlington MA) using a fluid-structure interaction physics. To simulate the bending deformation, the soft resistive sensor array embedded in PDMS was wrapped around the surface of the circular cylinders using a prescribed displacement and boundary loads applied to the outer faces of the PDMS (Supplementary Fig. 4). Figure 5c depicts the measured and simulated relative resistance changes Δ*R*/*R* for the soft resistive sensors with 500 nm and 1 μm line widths as a function of the bending radius. The resistance change upon bending can be understood by considering the effect of geometrical changes on the sensing resistor^29,56^. With decreasing bending radius, the measured Δ*R*/*R* for both 500-nm and 1-μm-wide straight-line resistors gradually increased by 7–8% by bending the resistor along the length direction and showed similar trends as the numerical simulation.

Similarly, Fig. 5d highlights a fabricated high-resolution, soft interdigitated capacitor with 5 μm line width and line spacing and up to 90 interdigitated electrodes (IDEs). Previously, soft interdigitated capacitors fabricated using a reverse stamping technique based on soft lithography were demonstrated with 50 μm line width, 100 μm line spacing, and up to 34 IDEs^29,56^. Figure 5e depicts the measured capacitance as a function of probing frequency up to 1 MHz for capacitors with different number of IDEs. The resulting capacitance was 0.2 pF in the case of 10 IDEs and linearly increased to 1.1 pF in case of the capacitor with 90 IDEs. Finite element simulations (COMSOL Multiphysics, COMSOL Inc., Burlington MA) were employed to simulate the capacitance using an electrostatic physics model (Supplementary Fig. 5). The measured capacitance for capacitors with different number of IDEs agrees within <15% with the simulated values.

Moreover, an all-soft microelectrode array (asMEA) with 100 electrodes, each 10 μm in diameter with 5-μm wide interconnections, was fabricated to highlight the high-density fabrication capability, as seen in Fig. 5f. The fabricated asMEA was then transferred to a PDMS substrate. Bending and twisting forces were applied to a single resistor, fabricated for a reliability test with the same dimensions (5 μm width, 1 μm thickness, and 1 mm length) as the asMEA microelectrodes (Supplementary Fig. 6). Figure 5g depicts the measured relative resistance change as a function of the number of bending (7.5 mm in radius) and twisting (180° twisting angle) cycles. The measured relative resistance changes are <±1% for up to 1000 bending and twisting cycles. Finally, commercial light-emitting diodes (LEDs) were integrated using a soft circuit and subjected to stretching and folding deformations, in order to demonstrate the flexibility and stretchability of the fabricated soft electronic components, as shown in Fig. 5h. Thanks to the intrinsically soft properties of EGaIn, the fabricated devices endure mechanical strain >30% as well as folding deformation, while maintaining their electrical functionality.

## Discussion

**Fig. 6:**
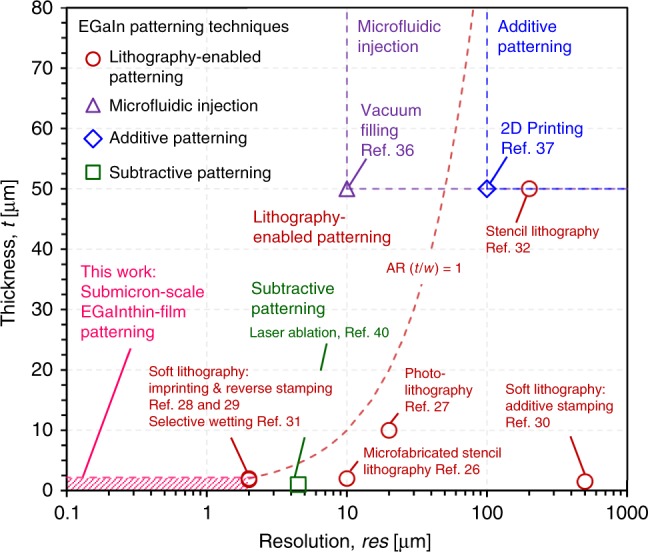
Liquid metal patterning technologies. Comparison of resolution (minimum line width) and film thickness of published liquid metal (eutectic gallium-indium alloy, EGaIn) patterning technologies as well as proposed nanofabrication technique based on hybrid lithography for submicron resolution.

This work presents a nanofabrication strategy for submicron-scale, all-soft electronic devices, and transducers based on EGaIn. Figure 6 shows a comparison of resolution and film thickness of published liquid metal patterning technologies, including lithography-enabled^26–32^, microfluidic injection^36^, and additive^37^ and subtractive^40^ patterning processes and highlights the resolution and film thickness range demonstrated using the nanofabrication strategy proposed in this work. Additive direct write and injection approaches enable simple, fast, and large-area EGaIn patterning. Direct writing techniques enable printing EGaIn patterns at desired locations, but their resolution (res) is limited to ≈ 100 μm with thicknesses (*t*) >50 μm because of the size limitation of the printing nozzles^37^. Microfluidic injection^34,35^ and vacuum filling^36^ approaches provide resolutions >10 μm, but the microchannels must have relatively large thicknesses >50 μm to reduce pressure drops, and their practical uses are limited when the EGaIn film needs to be exposed to the surface for additional processing. Subtractive and lithography-enabled patterning processes provide better resolution with wide-range patterning capabilities. Subtractive laser ablation enables to pattern fine EGaIn lines with res ≈ 5 μm and *t* < 1 μm;^40^ however, the serial process makes EGaIn removal slow when patterning small EGaIn features on large substrates. Patterning using lithography-defined stencils is simple and high-throughput and enables EGaIn structures on elastomeric substrates with res ≈ 10 μm and *t* ≈ 2 μm using microfabricated metal stencil films^26^ and res ≈ 20 μm and *t* ≈ 10 μm using photolithography and photoresist lift-off steps^27^. The limitations of this approach are the relatively low resolution, rough EGaIn surfaces, and considerable EGaIn waste during the stencil lift-off process. Soft lithography methods based on additive and subtractive reverse stamping processes enable wide-range EGaIn thin-film patterning from the single micrometer to the centimeter scale^28–30^. While micrometer-scale EGaIn thin-film patterning has been demonstrated, scaling these processes down to submicron features is difficult because of the high surface tension of EGaIn. Overall, patterning smooth and uniform EGaIn films with high-resolution and the ability to scale remains one of the primary technical hurdles for EGaIn-based soft electronic devices, as summarized in Supplementary Table 1.

The hybrid fabrication technique developed here to create submicrometer-scale EGaIn geometries is based on electron-beam lithography for micro/nanopattern definition in a PMMA electron-beam resist and soft lithography for EGaIn stamping. The nanofabrication technique was applied to a biphasic structure, comprising a metallic adhesion layer coated with EGaIn. The stamped EGaIn uniformly spreads across the Au or Cu thin films and fills concave nano/micropatterns in the underlying PMMA resist with EGaIn up to the chosen PMMA thickness. Submicron-scale EGaIn thin-film patterning with line width as small as 180 nm and line spacing as small as 1 μm was achieved, resulting in the highest resolution EGaIn patterning to date.

The developed nanofabrication process based on hybrid lithography shows competitive performance in terms of resolution, achievable wiring density, as well as strain limit, if compared to other types of stretchable conductors, namely serpentine solid metal and nanomaterial composite conductors. Serpentine solid metal patterns on a soft elastomer exhibit excellent electrical conductivity and can endure large mechanical strain. However, the necessary serpentine wires increase space requirements and, thus, lower the achievable wiring density compared to EGaIn wires with the same lateral resolution. On the other hand, nanoprinting techniques can create complex patterns with tens of nanometer resolution relying on high-resolution stamps fabricated using EBL or LIL processes. However, the printed nano patterns are not compliant structures, and the nanotransfer process is typically limited to print only on rigid substrates. Finally, printing conductive nanomaterials enables simple and inexpensive fabrication of conductors without the need for serpentine geometries. However, their limited resolution combined with the low electrical conductivity are major drawbacks. Considering this, intrinsically soft liquid metal patterned by the developed hybrid lithography technique offers a currently unrivaled combination of resolution, electrical conductivity, and resulting electronic/wiring density.

The demonstrated EGaIn-based submicron-scale and high-density soft passive electronic devices and sensor arrays fabricated using the hybrid lithography process are currently difficult if not impossible to build with other fabrication methods. Therefore, we anticipate that the developed EGaIn nanofabrication technique based on the hybrid lithography will open up exciting opportunities in the development of integrated soft electronic devices for applications in human organs-on-chips as well as skin- or body-integrated electronics. For example, considering the size of a single biological cell and its soft and dynamic properties, the demonstrated all-soft mechanotransducer array can be applied to integrated single-cell-level biological sensing platforms, possibly allowing to monitor disease progression and therapy. Similarly, the developed passive electronic devices can potentially be applied to all-soft and wireless pressure sensing devices based on RLC circuits for physiological pressure monitoring in the human body, such as intracranial pressure, intraocular pressure, cardiovascular pressure, and others.

## Methods

### Nano/microstructure fabrication process

A water-soluble sacrificial layer, poly(acrylic acid) (PAA, Polyscience, Inc.), was spun on a Si wafer at 2000 rpm for 30 s and baked at 100 °C for 60 s, resulting in ≈ 2 µm film thickness. On top of the PAA sacrificial layer, a parylene-C film with 600 nm thickness was deposited by chemical vapor deposition (CVD, SCS Labcoter PDS 2010). For nano/microstructure definition, electron-beam lithography (EBL, Elionix ELS G-100) was utilized to pattern spin-coated poly(methylmethacrylate) (PMMA, MicroChem Corp.) films with thicknesses ranging from 300 nm to 1 µm. In the EBL process, the samples with different PMMA thicknesses were all exposed using a 1-nA current with a proximity effect correction (*β* = 30 and *η* = 0.6). The applied dose was adjusted from 400 µC cm^−2^ to 630 µC cm^−2^ because of the different PMMA thicknesses. For example, a dose of 510 µC cm^−2^ was selected for the 1-μm-thick PMMA film. Then, a thin metallic adhesion layer, either Ti/Au or Ti/Cu, was deposited onto the PMMA nano/micropatterns using an electron-beam evaporator with a target thickness of 5 nm/30 nm.

### PDMS stamp preparation and EGaIn stamping process

For PDMS stamp fabrication, a general replica molding process was used using an acrylic master fabricated using a CO_2_ laser cutter (Hermes LS500XL). Liquid PDMS (10:1 ratio of PDMS pre-polymer and curing agent, Sylgard 184, Dow Corning) was drop-casted on the acrylic master and cured at 60 °C for 8 h. PDMS stamps with various shapes (e.g. circle or rectangle) and sizes (e.g. 5 mm ×5 mm to 30 mm ×30 mm) were designed and fabricated to stamp EGaIn onto the Au- or Cu-coated nano/microstructures.

EGaIn (gallium-indium eutectic, >%99.99 trace metal basis, Sigma-Aldrich) was dispensed on a donor PDMS substrate using a syringe and spread and flattened by a PDMS roller. In the next step, the fabricated PDMS stamp was wet with EGaIn by pressing it on the EGaIn-coated donor PDMS substrate and gently stamped 2–3 times onto the Au- or Cu-coated nano/micropatterns to transfer the EGaIn thin film. The stamped EGaIn on Au or Cu was finally patterned using a PMMA lift-off process with acetone.

### Soft material encapsulation and release process

The patterned EGaIn structures were encapsulated with liquid PDMS (10:1 ratio of PDMS pre-polymer and curing agent, Sylgard 184, Dow Corning) either by spin coating or drop casting. The fabricated soft electronic devices were then released by submerging the samples into water for >6 h. After the PAA sacrificial layer etching was completed, the fabricated soft devices were floating on the water surface and could gently be transferred to a glass substrate to etch the parylene-C layer. The parylene-C layer was etched using an oxygen plasma in a reactive-ion etching system (RIE, Vision 320 RIE) for >7 min or until the parylene-C film was completely removed. Under the etching conditions of 200 mTorr pressure and 200 W power, the tested parylene-C etch rate using oxygen plasma was ≈ 100 nm min^−1^. After etching the palylene-C layer, the soft electronic devices were encapsulated again with PDMS for backside sealing.

### Electrical and mechanical characterization, and optical measurement

Electrical testing of the soft passive components and circuits was performed using a multimeter (Hewlett Packard 34401A), a source meter (Keithley 2636A), and an LCR meter (Agilent 4284 A). All testing was performed at room temperature under ambient pressure. For bending characterization, different size circular glass cylinders (radius: 65, 32.5, 27.5, 13.5, and 7.5 mm) were used. High-resolution optical images of the patterned EGaIn structures were obtained using scanning electron microscopy (SEM, Hitachi S-3700N Variable Pressure SEM). 3D images and cross-sections were obtained using a laser confocal microscope (Olympus, LEXT OLS 4000).

## Supplementary information

Supplementary Information

## Data Availability

The data that support the findings of this study are available from the corresponding author upon reasonable request.
