# Supplementary material for: Nanofabrication for all-soft and high-density electronic devices based on liquid metal
Source: Nat Commun. 2020 Feb 21;11:1002. doi: 10.1038/s41467-020-14814-y (PMC7035367; doi:10.1038/s41467-020-14814-y)
Supplement: Supplementary file 1 — Supplementary Information [file 41467_2020_14814_MOESM1_ESM.pdf]

Supplementary Information

for

Nanofabrication for all-soft and high-density electronic devices based on liquid metal

M.-g. Kim et al.

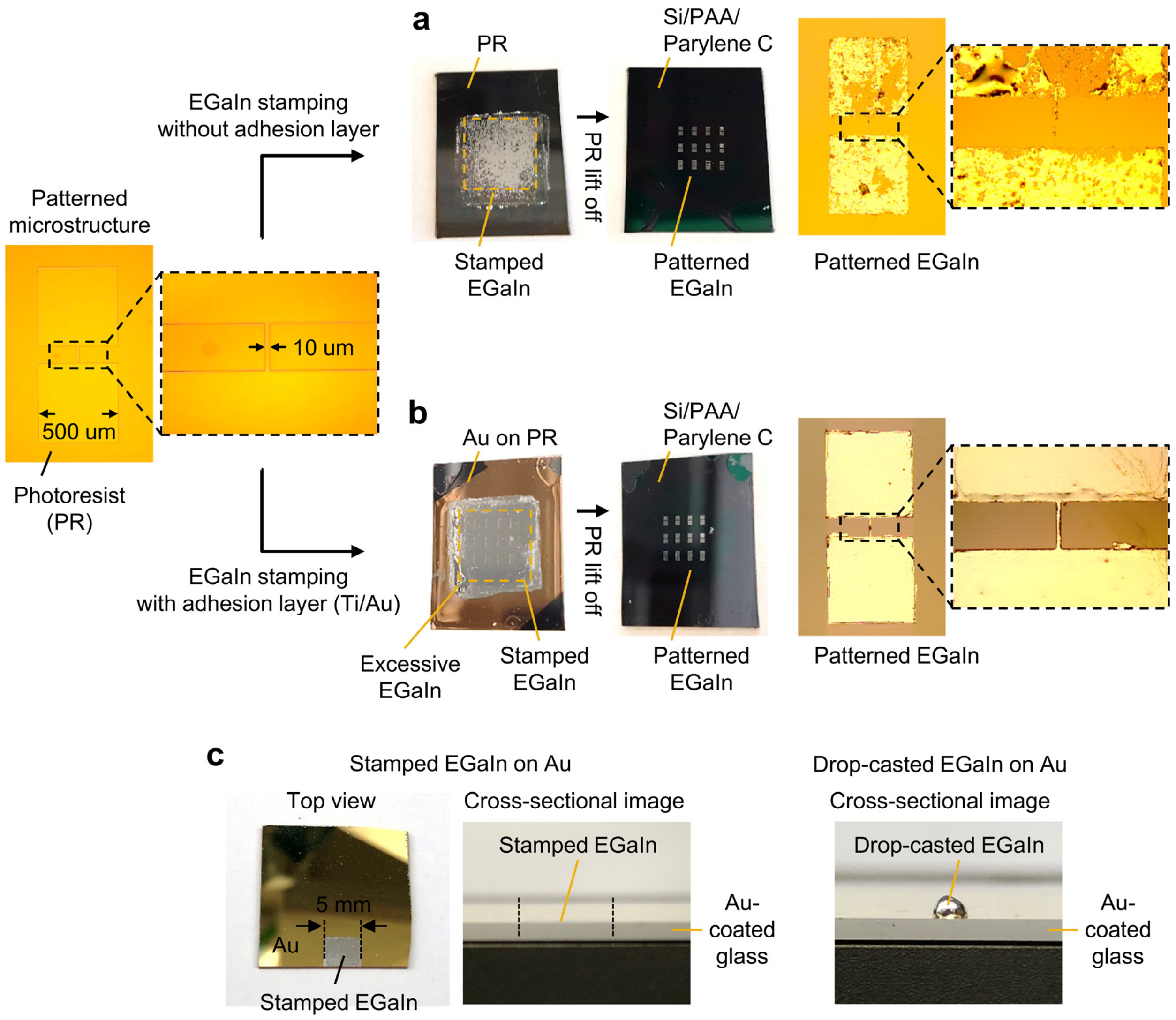

Supplementary Figure 1. Wetting characteristic of EGaIn stamping process. EGaIn stamping process on patterned PMMA structures **a** without adhesion layer and **b** with Au adhesion layer. **c** Cross-sectional images of uniformly spread stamped EGaIn on Au adhesion layer, in comparison with drop-casted EGaIn on the same Au adhesion layer. The contact angle of the EGaIn droplet on the Au surface is $\approx 130^{o}$[39,52]. The stamping process enables uniform deposition on the Au adhesion layer, resulting in a dramatically reduced contact angle of almost zero.

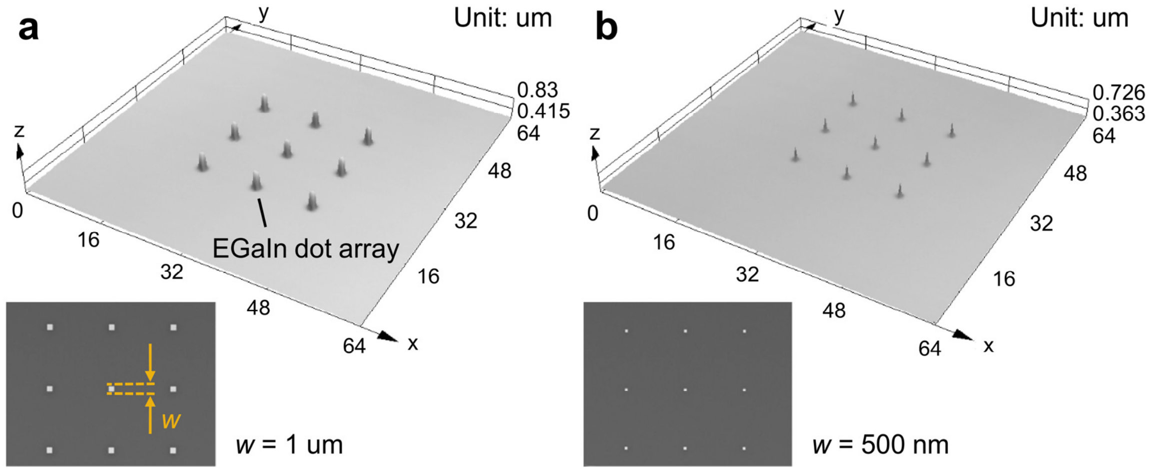

Supplementary Figure 2. Patterned EGaIn dot arrays. EGaIn dot array with dimensions of **a** 1 μm and **b** 500 nm.

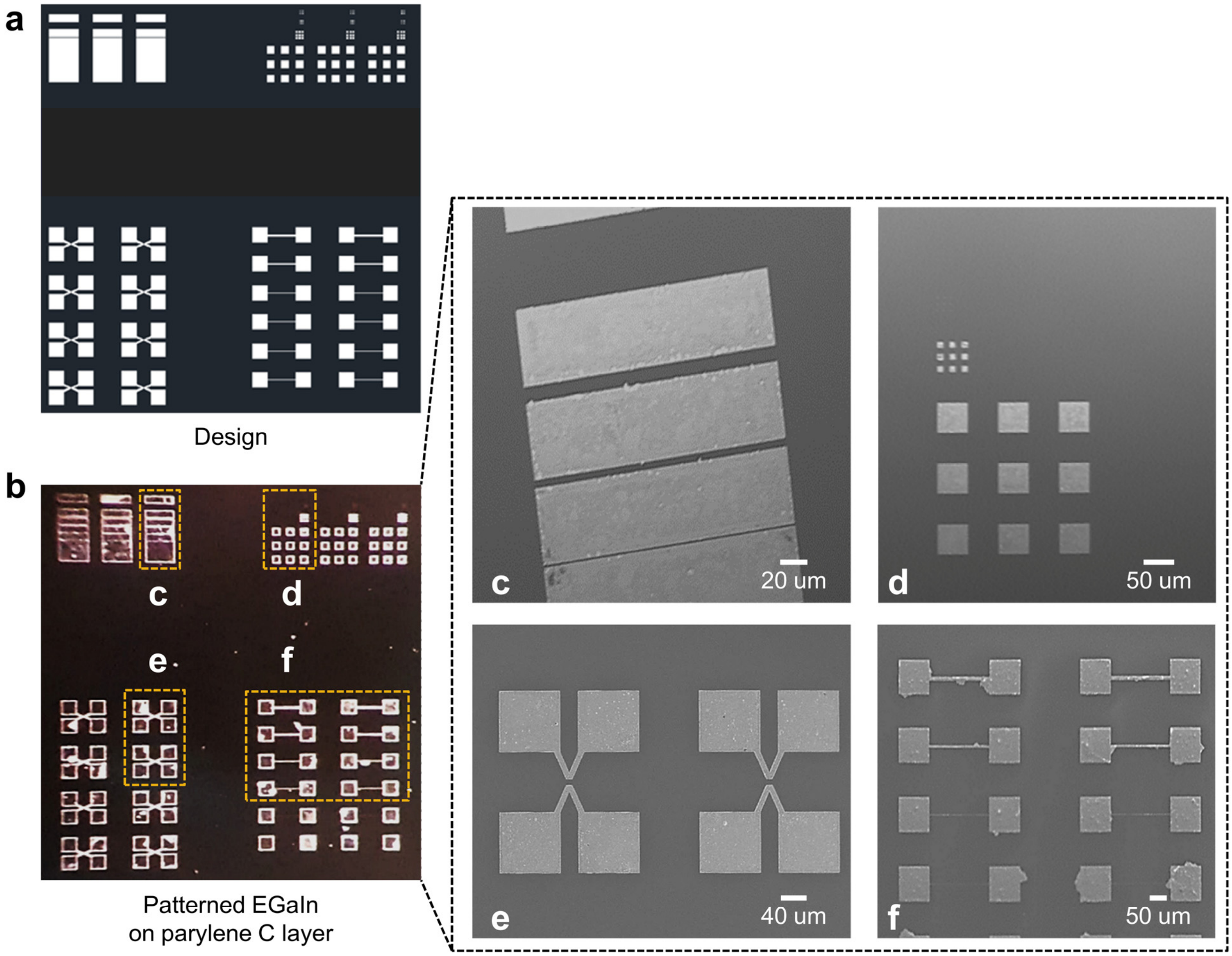

Supplementary Figure 3. Hybrid lithography process. **a** CAD design and **b** patterned EGaIn nano/microstructures on Si wafer with detailed SEM images, including **c** test structures for line spacing, **d** square-shaped dot arrays, **e** electrical test structures for resolution characterization, and **f** resistor test structures with different line widths.

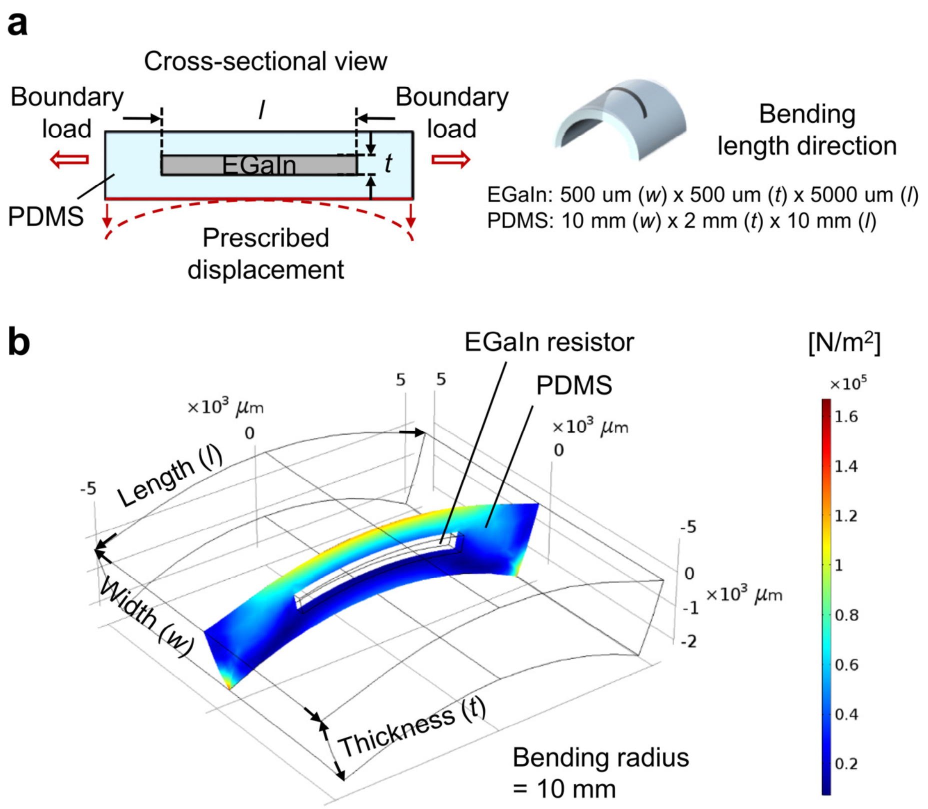

Supplementary Figure 4. Numerical evaluation of bending deformation of soft resistive sensor. **a** schematic of simulation model with applied boundary conditions[23] and **b** resulting finite element simulation of bending over a 10 mm radius circular cylinder along the length direction using COMSOL Multiphysics (COMSOL Inc., Burlington MA).

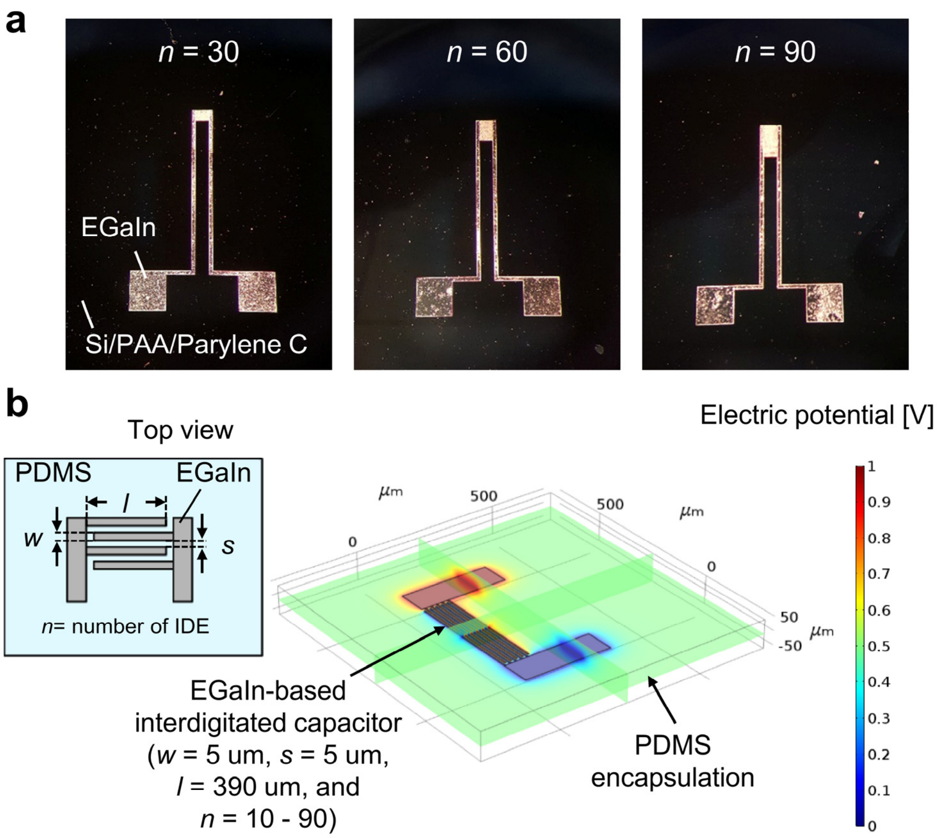

Supplementary Figure 5. Numerical evaluation of EGaIn-based interdigitated capacitor. **a** Fabricated EGaIn-based interdigitated capacitors with different number n of interdigitated electrodes (IDE). **b** Schematic of simulation model and simulated electric potential distribution for interdigitated capacitor with an applied voltage of 1 V using COMSOL Multiphysics (COMSOL Inc., Burlington MA).

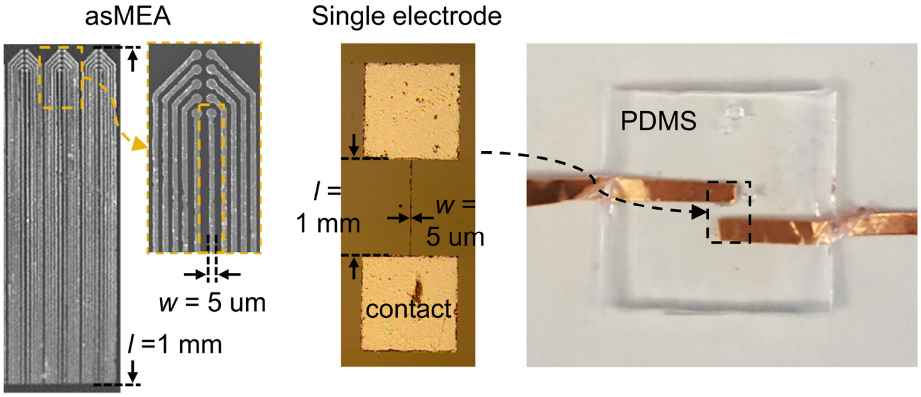

Supplementary Figure 6. Fabricated all-soft microelectrode array. Fabricated microelectrode array with 100 electrodes and fabricated single electrode with same geometries (width (w) = 5 μm and length (l)= 1 mm) for bending and twisting tests.

Supplementary Table 1. Summary of EGaIn patterning methods using lithography-enabled, injection, additive, and subtractive processes and comparison with the proposed nanofabrication technique based on hybrid lithography.

**Patterning Methods**	**Approach**	**Resolution (*w*)**	**Spacing (*s*)**	**Thickness (*t*)**	**Note**
Lithography -enabled process	**Hybrid lithography (This work)**	**180 nm**	**1 µm**	**300 nm - 1 µm**	– **Submicron-scale resolution**<br>– **Thin-film fabrication**<br>– **Complicated and multi-step process**<br>– **Expensive fabrication**
	Soft lithography: imprinting[28] & reverse stamping[29]	2 µm	1-2 µm	< 2 µm	– Micrometer-scale resolution<br>– Thin-film fabrication<br>– Uniform surface
	Additive stamping[30]	500 µm	500 µm	≈ 1.5 µm	– Simple and easy process<br>– Large-area patterning > cm<br>– Thin-film fabrication<br>– Uniform surface
	Metal stencil film[32]	200 µm	100 µm	6-30 µm	– Simple and high throughput process<br>– Relatively large feature size<br>– Non-uniform thickness<br>– Rough edges
	Microfabricated metal stencil film[26]	10 µm	10 µm	≈ 2 µm	
	Photo Lithography[27]	20 µm	40 µm	≈ 10 µm	
Injection	Fluidic injection[34]	70 µm	>70 µm	70 µm	– Simple, fast, and low-cost process<br>– Uniform thickness, but requires high channel thickness
	Vacuum filling[36]	≈ 10 µm	-	> 50 µm	
Additive process	2D printing[37]	≈ 100 µm	-	> 50 µm	– 2D/3D structure fabrication<br>– Relatively low resolution<br>– Uniform thickness
	3D printing[38]	≈ 100 µm	-	≈ 100 µm	
	2D printing and transfer [39]	2 µm	-	> 80 µm	– Micrometer-scale resolution<br>– Limited design<br>– Complicated and multi-step process
Subtractive process	Laser[40]	4.5 µm	100 µm	< 1 µm	– Micrometer-scale resolution<br>– Serial process
	Electrochemical reduction[44]	≈ mm	-	≈ 120 µm	– Inexpensive and facile process<br>– Relatively low resolution<br>– Non-uniform thickness
